# Randomized Controlled Trial of Oral Vancomycin Treatment in Clostridioides difficile-Colonized Patients

**DOI:** 10.1128/mSphere.00936-20

**Published:** 2021-01-13

**Authors:** Skye R. S. Fishbein, Tiffany Hink, Kimberly A. Reske, Candice Cass, Emily Struttmann, Zainab Hassan Iqbal, Sondra Seiler, Jennie H. Kwon, Carey-Ann D. Burnham, Gautam Dantas, Erik R. Dubberke

**Affiliations:** a The Edison Family Center for Genome Sciences and Systems Biology, Washington University School of Medicine, St. Louis, Missouri, USA; b Department of Pathology and Immunology, Washington University School of Medicine, St. Louis, Missouri, USA; c Division of Infectious Diseases, Washington University School of Medicine, St. Louis, Missouri, USA; d Department of Molecular Microbiology, Washington University School of Medicine, St. Louis, Missouri, USA; e Department of Pediatrics, Washington University School of Medicine, St. Louis, Missouri, USA; f Department of Biomedical Engineering, Washington University School of Medicine, St. Louis, Missouri, USA; University of Michigan-Ann Arbor

**Keywords:** *C. difficile*, vancomycin-resistant enterococci, vancomycin

## Abstract

Clostridioides difficile infection (CDI) is most commonly diagnosed using nucleic acid amplification tests (NAAT); the low positive predictive value of these assays results in patients colonized with C. difficile unnecessarily receiving CDI treatment antibiotics. The risks and benefits of antibiotic treatment in individuals with such cases are unknown. Fecal samples of NAAT-positive, toxin enzyme immunoassay (EIA)-negative patients were collected before, during, and after randomization to vancomycin (*n* = 8) or placebo (*n* = 7). C. difficile and antibiotic-resistant organisms (AROs) were selectively cultured from fecal and environmental samples. Shotgun metagenomics and comparative isolate genomics were used to understand the impact of oral vancomycin on the microbiome and environmental contamination. Overall, 80% of placebo patients and 71% of vancomycin patients were colonized with C. difficile posttreatment. One person randomized to placebo subsequently received treatment for CDI. In the vancomycin-treated group, beta-diversity (*P = *0.0059) and macrolide-lincosamide-streptogramin (MLS) resistance genes (*P = *0.037) increased after treatment; C. difficile and vancomycin-resistant enterococci (VRE) environmental contamination was found in 53% of patients and 26% of patients, respectively. We found that vancomycin alters the gut microbiota, does not permanently clear C. difficile, and is associated with VRE colonization/environmental contamination. (This study has been registered at ClinicalTrials.gov under registration no. NCT03388268.)

**IMPORTANCE** A gold standard diagnostic for Clostridioides difficile infection (CDI) does not exist. An area of controversy is how to manage patients whose stool tests positive by nucleic acid amplification tests but negative by toxin enzyme immunoassay. Existing data suggest most of these patients do not have CDI, but most are treated with oral vancomycin. Potential benefits to treatment include a decreased risk for adverse outcomes if the patient does have CDI and the potential to decrease C. difficile shedding/transmission. However, oral vancomycin perturbs the intestinal microbiota and promotes antibiotic-resistant organism colonization/transmission. We conducted a double-blinded randomized controlled trial to assess the risk-benefit of oral vancomycin treatment in this population. Oral vancomycin did not result in long-term clearance of C. difficile, perturbed the microbiota, and was associated with colonization/shedding of vancomycin-resistant enterococci. This work underscores the need to better understand this population of patients in the context of C. difficile/ARO-related outcomes and transmission.

## Abstract (precis)

A gold standard diagnostic for Clostridioides difficile infection (CDI) does not exist. An area of controversy is how to manage patients whose stool tests positive by nucleic acid amplification tests but negative by toxin enzyme immunoassay.

## INTRODUCTION

The CDC estimates there were 223,900 cases of Clostridioides difficile infection (CDI) requiring hospitalization in 2017 in the United States (1). Previous antibiotic use, chemotherapy, and an extended stay in a health care facility are associated with CDI (2). Concomitantly, such risk factors are associated with colonization by antibiotic-resistant organisms (AROs), which cause significant mortality and burden on the health care system (3, 4). Interventions such as increasing antibiotic stewardship and decreasing organism transmission are associated with reductions in CDI incidence and ARO infections (5).

Diagnosis of CDI remains a significant challenge, as C. difficile can cause a spectrum of illnesses in patients, from asymptomatic colonization to diarrhea, fulminant colitis, and death (6). Initially, toxin enzyme immunoassays (EIAs) were the most commonly used diagnostic assay, but they suffered from low analytical sensitivity (7). In response, nucleic acid amplification tests (NAATs) that detect C. difficile toxin DNA in stool were developed with increased analytical sensitivity compared to that of toxin EIAs. Due to this enhanced sensitivity, NAAT assays are more likely than EIAs to detect C. difficile colonization in the absence of CDI (8). In turn, a significant proportion of patients whose stool tests EIA negative/NAAT positive (EIA^−^/NAAT^+^) are considered asymptomatic carriers, with diarrhea from other causes (9). When NAATs are used for diagnostic testing, most patients with NAAT-positive stool receive treatment for CDI (10).

Oral vancomycin is a first-line, widely accepted treatment for CDI (11). While there is a clear benefit to administering oral vancomycin when a patient has CDI, the risk-benefit balance of oral vancomycin when administered to EIA^−^/NAAT^+^ patients is less clear. If the EIA is falsely negative for CDI, one benefit of treating EIA^−^/NAAT^+^ patients is the potential avoidance of CDI-associated adverse outcomes, such as sepsis, toxic megacolon, and death (12). Oral vancomycin has been found to reduce shedding of C. difficile in feces of colonized patients (13), potentially reducing contamination of their hospital environment (environmental contamination) and C. difficile transmission to other patients. However, antibiotics disturb the gut microbiota, increase the risk of CDI, and also can affect the composition of resistance gene determinants (the resistome) in the gut microbiota (14–16). Further, oral vancomycin is associated with colonization and expansion of vancomycin-resistant enterococci (VRE) in CDI-treated patients (17, 18). These expansions may increase the risk of subsequent infection in the patient as well as environmental contamination by the colonized patient and subsequent transmission.

In this study, we performed a double-blinded randomized control trial to examine the effect of oral vancomycin on C. difficile-colonized patients (EIA^−^/NAAT^+^). Specifically, we analyzed patient gut communities over time to understand the extent of microbiome and resistome alterations correlated with vancomycin treatment. Additionally, we examined the influence of this treatment on C. difficile and ARO environmental contamination.

## RESULTS

### Study population and sample collection.

**FIG 1:**
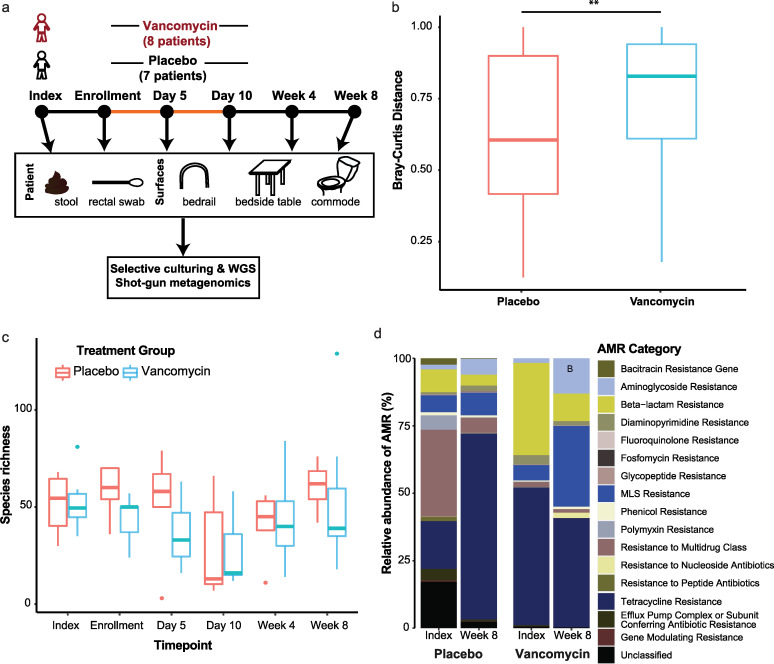
Vancomycin effect on C. difficile-colonized patient gut microbiomes. (a) Randomized control trial to test the effect of 10 days of oral vancomycin treatment on health-related outcomes in C. difficile-colonized patients. Patient stool and surfaces were sampled to examine patient microbiomes and environmental contamination of hospital environments. (b) Beta-diversity, as measured by Bray-Curtis dissimilarity, distributions of within-patient comparisons between placebo and vancomycin treatment groups. Dissimilarity was significantly different between treatment groups (**, *P* = 0.0057) as measured by a Wilcoxon rank sum test. (c) Measurement of alpha-diversity (richness) of microbial species in patient fecal samples over time due to vancomycin treatment. Richness was not significantly affected by vancomycin treatment (*P* = 0.23), as examined by a two-way analysis of variance. (d) Relative abundance of major antibiotic resistance (AMR) classes before (index) and after (week 8) treatment, averaged across patients. MLS, macrolide-lincosamide-streptogramin.

The charts of 3,089 patients whose stool tested EIA^−^ were reviewed, and of those patients, 648 were eligible for NAAT testing. Of these, 65 (10%) were NAAT^+^, and 15 were enrolled. Eight were randomized to receive oral vancomycin (see Fig. S1 in the supplemental material). The majority (80%) of patients had previous hospitalizations (Table 1). At enrollment and during treatment, 71% of patients in the placebo group received nonstudy antibiotics relative to 88% in the vancomycin group (Fig. S2a). Fecal samples (or rectal swabs if fecal samples were not available) and environmental samples were collected from patients and their environments (Fig. 1a), and the microbiomes were analyzed for patients with specimens from at least three time points (Text S1). Therefore, patients 4, 5, and 6 were excluded.

**TABLE 1:** Patient demographics^*a*^

Demographic element	Value for:	
	Placebo (*n* = 7)	Vancomycin (*n* = 8)
Receiving antibiotics at enrollment [no. (%)]	2 (29)	3 (38)
Age [yr; median (range)]	64 (48–77)	66 (37–81)
Female [no. (%)]	6 (86)	4 (50)
Nonwhite race [no. (%)]	1 (14)	1 (13)
Comorbidities [no. (%)]		
Diabetes mellitus	3 (43)	2 (25)
Cancer (excluding leukemia/lymphoma)	2 (29)	4 (50)
Leukemia/lymphoma	4 (57)	3 (38)
Irritable bowel syndrome	1 (14)	1 (13)
Chemotherapy at enrollment or in previous 4 weeks [no. (%)]	5 (71)	4 (50)
Laxative within 48 h of stool collection [no. (%)]	2 (29)	3 (38)
Laxative within 48 h of study enrollment [no. (%)]	2 (29)	3 (38)
Hospitalization(s) in previous year [no. (%)]	6 (86)	6 (75)
No. of previous hospitalizations [no. (%)]		
0	1 (14)	2 (25)
1 or 2	2 (29)	3 (38)
3 or more	4 (57)	3 (38)

a Patients were randomized based on whether or not they were receiving antibiotics at enrollment.

10.1128/mSphere.00936-20.1 FIG S1 Screening enrollment flow chart for double-blinded, randomized controlled Trial of Oral vancomycin versus placebo in hospitalized patients with diarrhea and stool toXin NEGative but nucleic acid amplification test positive for toxigenic Clostridioides difficile. EIA, enzyme immunoassay; NAAT, nucleic acid amplification test; SNF, skilled nursing facility FIG S1, PDF file, 0.7 MB. Copyright © 2021 Fishbein et al. 2021 Fishbein et al. This content is distributed under the terms of the Creative Commons Attribution 4.0 International license.

10.1128/mSphere.00936-20.2 FIG S2 **(**a) Timeline of patient antimicrobial use. The gray zone indicates the timezone from study enrollment to week 8 for each patient. Amp/Sul, ampicillin-sulbactam; Pip/Tazo, piperacillin-tazobactam; TMP/SMX, trimethoprim-sulfamethoxazole. (b) Relative abundance of microbial taxa at the level of order for a set of patient-time point comparisons, where grouped bar plots indicate microbiomes from different sample types at the same patient-time point. RS, rectal swab; ST, stool. (c) Bray-Curtis dissimilarity distributions for comparisons between microbiomes from the same patient-time point (rectal swab-stool), from different time points within one patient (within patient), and from different patients (between patients). Distances were significantly different between rectal swab-stool and within-patient (****, *P* = 4.2e−9) comparisons, between rectal swab-stool and between patient (****, *P* = 4.2e−9), and between within patient and between patient (****, *P* < 2e−16), as determined by a pairwise Wilcoxon test with a Bonferroni multiple testing correction. (d) Relative abundances of C. difficile in patient microbiomes over time; patient 12 is depicted (green, thick line). Download FIG S2, PDF file, 0.8 MB. Copyright © 2021 Fishbein et al. 2021 Fishbein et al. This content is distributed under the terms of the Creative Commons Attribution 4.0 International license.

10.1128/mSphere.00936-20.5 TEXT S1 Extended discussion of study methods and results. Download Text S1, DOCX file, 0.03 MB. Copyright © 2021 Fishbein et al. 2021 Fishbein et al. This content is distributed under the terms of the Creative Commons Attribution 4.0 International license.

### C. difficile-related outcomes and colonization poststudy.

One patient (vancomycin group) had new/worsening abdominal pain, while one patient’s stool (placebo group) tested positive by toxin EIA (described below). With the CDI-related events described above, 5 patients in total had adverse events, including hypoxemia, vertigo, and altered mental status. The median time to end of clinically significant diarrhea (CSD) was 4 days in the placebo group and 2 days in the vancomycin group (*P = *0.46). Posttreatment, 80% of placebo patients and 71% of vancomycin-treated patients had stool containing culturable C. difficile.

Patient 12 (placebo group) had persistent diarrhea, and the treating physician ordered repeat C. difficile testing, which returned EIA^+^ at the S02 time point. The study blind was broken and the patient was started on oral vancomycin. To better understand this patient’s trajectory, a more thorough investigation of this patient was conducted.

The patient had been admitted for autologous hematopoietic cell transplantation (HCT) for lymphoma. The patient developed neutropenia, mucositis, and diarrhea when expected based on the HCT conditioning regimen received (carmustine, etoposide, aracytin, and melphalan). This prompted stool collection for C. difficile testing that became the qualifying stool specimen (S00). The diarrhea persisted into the trial, leading the treating clinicians to order repeat testing for C. difficile, and the stool was EIA^+^ at day 5. Her diarrhea started to improve 2 days after CDI treatment was started; this also corresponded to neutrophil recovery and improvement in mucositis. Examination of C. difficile levels in the fecal metagenomics revealed that at time S00, patient 12 had 0.15% C. difficile abundance, while the whole patient population had 0 to 1.3% C. difficile abundance (Fig. S2d). At S02, patient 12 had 1.5% C. difficile abundance (0 to 1.5% C. difficile abundance in total population).

### Vancomycin effect on gut microbiome and resistome.

Patient’s microbiomes shifted more significantly in the vancomycin group, as measured by within-patient beta-diversity (Fig. 1b; *P = *0.005). Alpha-diversity decreased in both groups during treatment; vancomycin did not significantly alter alpha-diversity relative to that of the placebo (Fig. 1c). We also examined treatment-related perturbations to the gut resistome in a subset of patients (three from each group) using ShortBred to map metagenomic reads to known antibiotic resistance genes (ARGs). We examined changes in the abundance of ARG class markers between the treatment groups (before, S00, and after, S05, treatment) and found that the class of multidrug resistance (MDR) markers decreased by 25% (*P = *0.0071) in the placebo group, the class of genes encoding efflux pump machinery decreased by 55% (*P < *0.0001) in the placebo group, while the class of genes encoding macrolide-lincosamide-streptogramin (MLS) resistance increased by 22% (*P = *0.037) in the vancomycin group (Fig. 1d). Finally, the relative alpha-diversity of ARGs was significantly increased after treatment in the vancomycin group (Fig. S3a; *P = *0.028).

**FIG 2:**
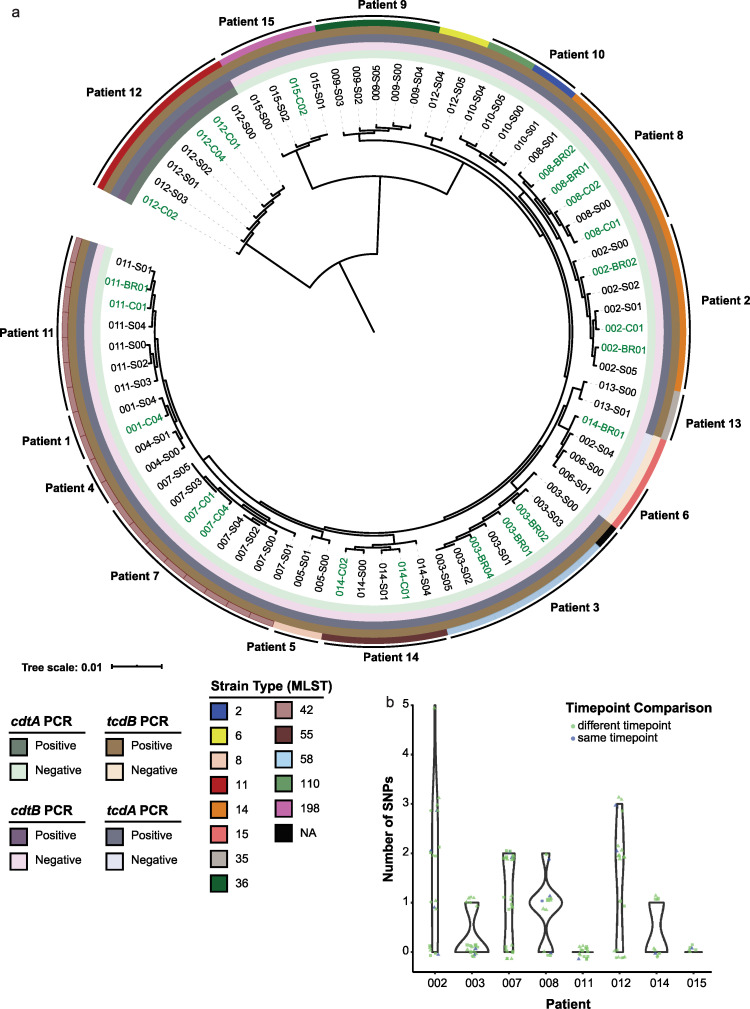
Patient shedding of C. difficile associated with environmental contamination. (a) Approximate maximum-likelihood tree of 75 C. difficile genomes isolated from patient stool and their environment. Each node represents an isolate found from a patient-time point, where the number before the dash represents the patient and letter after the dash represents the source of the isolate (S, stool; BR, bedrail; C, commode), followed by the number representing the time point of isolation. Isolates colored green represent those recovered from the environment. Color strips represent the outcome of NAAT testing for C. difficile toxins (*tcdAB* and *cdtAB*) and *in silico* MLST typing. (b) Distribution of pairwise single-nucleotide polymorphism (SNP) distances for each patient-isolate group. Distances are visually classfied in two ways: by time point, either between time point comparisons (green) or within time point comparisons (blue), and by source, either environmental-only comparisons (●), stool-to-environment comparisons (▲), or stool-only comparisons (■).

10.1128/mSphere.00936-20.3 FIG S3 (a) Measurement of relative alpha-diversity, or Shannon diversity, for ARG compositition in a subset of patients on vancomycin (blue) or placebo (red). Time points correspond to before (index), during (day 10), and after (week 8) treatment. Shannon diversity was significantly different between treatment groups (*P = *0.028) at week 8, as examined by a Welch’s two-sample *t* test. (b) Approximate-maximum-likelihood tree of 250 C. difficile genomes. ST11 strains are colored green if they were isolated from this cohort and blue if they were previously published ST11 genomes. (c) SNP distances determined from the core-genome alignment of the isolate cohort visualized in Fig. 2a. ****, *P *< 0.0001, as determined by pairwise Wilcoxon rank sum test, corrected for multiple testing using Bonferroni. Download FIG S3, PDF file, 0.8 MB. Copyright © 2021 Fishbein et al. 2021 Fishbein et al. This content is distributed under the terms of the Creative Commons Attribution 4.0 International license.

### Phylogenetic examination of C. difficile to quantify environmental contamination.

In addition to defining any effects of vancomycin on C. difficile-related outcomes and the microbiome, we examined the C. difficile isolate population to make inferences about pathogenicity and patient shedding. We sequenced 75 C. difficile isolates cultured from patient stools and their environment. A maximum-likelihood tree of the core genome (consisting of 2,628 genes) alignment of our isolates displayed patient-specific clades, indicating that most patients harbored one dominant isolate (Fig. 2a); multilocus sequence typing (MLST) analysis confirmed that 67% of patient-isolate groups were defined by one sequence type (ST). Notably, patient 12’s ST11 isolates were the only isolates to test positive for the toxin genes *cdtA* and *cdtB* by multiplex PCR (Fig. 2a and Table S2) (19). To contextualize our cohort, we used 250 C. difficile genomes representing all known strain types and our 75 isolate genomes to generate a core genome alignment (consisting of 1,961 genes) and computed a maximum-likelihood phylogenetic tree. In both phylogenetic trees, ST11 isolates (from patient 12 and previously published) formed a distinct clade from other isolates (Fig. S3b).

10.1128/mSphere.00936-20.6 TABLE S1 ToxNeg study organization. Patient-time point sample information with metadata. Download Table S1, XLSX file, 0.01 MB. Copyright © 2021 Fishbein et al. 2021 Fishbein et al. This content is distributed under the terms of the Creative Commons Attribution 4.0 International license.

10.1128/mSphere.00936-20.7 TABLE S2 C. difficile isolate list. Isolates collected from patient and environment with corresponding strain information. Download Table S2, XLSX file, 0.01 MB. Copyright © 2021 Fishbein et al. 2021 Fishbein et al. This content is distributed under the terms of the Creative Commons Attribution 4.0 International license.

To identify instances of environmental contamination with C. difficile by patients, we determined strain relatedness (single-nucleotide polymorphism [SNP] distances) between stool and environmental isolates in two ways. First, from the core genome alignment (Fig. 2a), we found that SNP distances between isolates from the same patient-time point (*P < *0.0001) and from the same patient (*P < *0.0001) were significantly lower than between-patient pairwise SNP distances (Fig. S2c). In the second approach, quantification of pairwise SNP distances from patient-specific pseudoreference assemblies revealed that all isolates within a patient were less than 6 SNPs apart from one another (Fig. 2b and Table S3). Fifty-three percent of patients had evidence of environmental contamination with a nearly identical clone (≤2 SNPs) to their corresponding stool isolate. These data indicate that colonized C. difficile patients serve as a source of C. difficile contamination of the hospital environment, regardless of vancomycin treatment.

10.1128/mSphere.00936-20.8 TABLE S3 C. difficile SNP distances. Pairwise C. difficile isolate comparisons using two different methods. Download Table S3, XLSX file, 0.08 MB. Copyright © 2021 Fishbein et al. 2021 Fishbein et al. This content is distributed under the terms of the Creative Commons Attribution 4.0 International license.

### ARO colonization of patients and environment.

Because patients at risk for CDI are also susceptible to ARO colonization/infection, we were interested in identifying the AROs that exist in the patients and in their environment. We collected 57 AR-*Enterobacterales* isolates, 29 VRE isolates, 14 *Pseudomonas* isolates, and 17 other ARO isolates from cultures of patient stool and the environment. Of the 117 isolates collected, 84 were recovered from patient stool and 33 were recovered from the environment. We found the number of ARO isolates recovered from any patient/patient environment varied across patients (Table S4, Fig. S4a). Antimicrobial susceptibility testing (AST) of 56 *Enterobacterales* isolates from patients’ stools revealed that 46% were resistant to ciprofloxacin, 7% were resistant to cefepime, and 23% were resistant to piperacillin-tazobactam. Sixty-seven percent of patients were colonized by at least one AR-*Enterobacterales* isolate (Fig. S4b).

10.1128/mSphere.00936-20.4 FIG S4 (a) Number of isolates associated with each patient during the trial that were C. difficile (CD), *Pseudomonas* spp., vancomycin-resistance enterococci (VRE), or *Enterobacterales*. (b) Antimicrobial susceptibility testing (AST) data for *Enterobacterales* isolates collected from patient stools, where resistance status was determined in accordance with the Clinical and Laboratory Standards Institutes (CLSI) guidelines. The legend above the clustogram indicates the patient, corresponding treatment group, and time point of the isolate. TMP/SMX, trimethoprim-sulfamethoxazole. (c) Composition of genetic resistance elements identified in VRE isolate cohort using AMRfinder. Each row represents summation of abundances of all antibiotic resistance genes (ARGs) in a given resistance class. (d) Relative abundance of E. faecium for all patients with detectable levels, from fecal microbiome measurements shown in Fig. 1. Circles indicate corresponding isolation of VRE E. faecium at the same time point from the patient’s stool or environment. Download FIG S4, PDF file, 0.3 MB. Copyright © 2021 Fishbein et al. 2021 Fishbein et al. This content is distributed under the terms of the Creative Commons Attribution 4.0 International license.

### VRE colonization and patient shedding.

**FIG 3:**
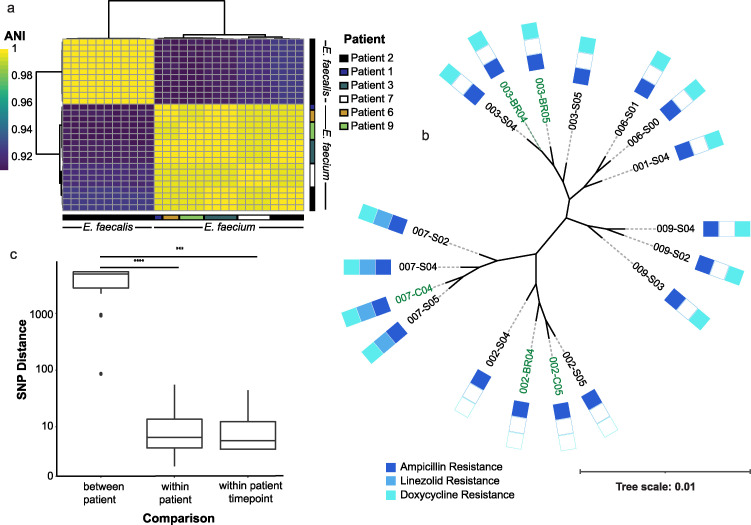
E. faecium isolates associated with VRE patient shedding/environmental contamination. (a) Pairwise average nucleotide identity (ANI) clustogram between *Enterococcus* isolates, where the color of the box indicates ANI between two isolate genomes. (b) Approximate maximum-likelihood phylogenetic tree of E. faecium isolates. Colored boxes indicate antimicrobial susceptibility testing (AST), where resistance status was determined in accordance with the Clinical and Laboratory Standards Institute (CLSI) guidelines. (c) Pairwise SNP distances derived from the core genome alignment. *P* values were generated through a Wilcoxon rank sum test. ****, <0.0001; ***, <0.001.

Treatment of C. difficile patients with oral vancomycin can facilitate VRE colonization in the patient (20). Of the five patients with VRE isolated from posttreatment time points (S04 and/or S05), three were in the vancomycin group. Visualization of average nucleotide identity (ANI) across VRE isolates (Fig. 3a) confirmed matrix-assisted laser desorption ionization–time of flight mass spectrometry (MALDI-TOF-MS) identification of isolates (21), revealing that E. faecium was isolated from the stool/environment of 7 patients, and E. faecalis was only isolated from the stool/environment of patient 2 (vancomycin group). For E. faecium isolates, we aligned core genomes (consisting of 2,115 genes) and constructed a maximum-likelihood phylogenetic tree. Patient-specific clades corresponded to distinct patterns of phenotypic resistance to ampicillin, doxycycline, and linezolid (Fig. 3b). Genomic ARG analysis indicated that all enterococcal isolates had the essential components of a functional *van* operon (*vanR-vanS-vanH-vanA-vanX*) (22), with E. faecalis possessing distinct ARGs relative to E. faecium isolates (Fig. S4c).

### Environmental contamination via patient shedding of VREs.

We measured SNP distance as described above to understand patient shedding of clones into the environment. SNP distances from the core genome alignment (Fig. 3c) indicated that patient-environment isolates from the same time point had significantly lower SNP distances than within-patient distances (*P = *0.001) or between-patient distances (*P < *0.0001). Using pseudoreference assemblies for each patient, all pairwise SNP distances within each patient were ≤8 SNPs (Table S5). Two patients in the vancomycin group shed VRE clones into their environment (0 SNPs between a stool and environmental isolate from the same time point). Additionally, in vancomycin patients (2, 3, and 9), the metagenomic relative abundance of E. faecium peaked at time points where highly related stool/environmental isolates were recovered (Fig. S4d).

10.1128/mSphere.00936-20.9 TABLE S4 ARO isolate and drug susceptibility data. List of ARO isolates collected from patients and environment with corresponding AST data. Download Table S4, XLSX file, 0.03 MB. Copyright © 2021 Fishbein et al. 2021 Fishbein et al. This content is distributed under the terms of the Creative Commons Attribution 4.0 International license.

10.1128/mSphere.00936-20.10 TABLE S5 VRE SNP distances. Pairwise VRE isolate comparisons using two different methods. Download Table S5, XLSX file, 0.01 MB. Copyright © 2021 Fishbein et al. 2021 Fishbein et al. This content is distributed under the terms of the Creative Commons Attribution 4.0 International license.

## DISCUSSION

We examined the risk-benefit of treating EIA^−^/NAAT^+^ patients with oral vancomycin by tracking C. difficile-related outcomes, gut microbiota changes, and environmental contamination by C. difficile and AROs in a double-blinded randomized control trial. We observed appreciable differences in the gut microbiome and resistome due to vancomycin, characterized the development of one EIA^+^ case, and identified multiple instances of C. difficile and VRE environmental contamination.

Vancomycin treatment resulted in an increase in beta-diversity within the gut microbiome but did not significantly alter alpha-diversity (species richness) during the trial relative to the placebo group. Interestingly, the median richness in the vancomycin-treated group at day 5 is lower than that of the placebo group, although this difference was not statistically significant. We predict that with an increased sample size, it is possible that we would have the statistical power to detect a difference in alpha-diversity between the treatment groups. More importantly, clinical data concerning the nonstudy trial antibiotics confirm that the placebo group was receiving antibiotics during the trial, perhaps obscuring vancomycin-related changes in alpha-diversity. However, the vancomycin-related perturbations that we did observe, despite high antibiotic exposure, suggest that this antibiotic has a more profound effect on the microbiome than that measured here.

A purported benefit of vancomycin treatment of colonized patients is reduction in C. difficile burden and, thus, a subsequent reduction in environmental contamination and transmission. Oral vancomycin treatment did not affect poststudy colonization with C. difficile; the majority of patients in both groups had viable C. difficile in their stool. In addition, we found evidence of environmental contamination in both patient groups. Given that vancomycin is also a known risk factor for CDI (23), our study does not support the notion that oral vancomycin could provide lasting reductions in C. difficile burden or transmission.

Patient 12 exemplifies the quandary in managing EIA^−^/NAAT^+^ patients and the reason this study was conducted. There are three possibilities to explain patient 12: (i) the first EIA was falsely negative for CDI; (ii) the first EIA was a true negative for CDI and the second EIA was a true positive for CDI; and (iii) the second EIA was a false positive for CDI. A detailed review of the clinical, genomics, and metagenomics data demonstrates the challenges in making these distinctions. Metagenomics data revealed an increase in C. difficile burden by S02, supporting a change from EIA^−^ to EIA^+^. Whole-genome sequencing (WGS) data identified the patient’s stool C. difficile isolates as an ST11 strain. This strain contains the accessory binary toxin locus, which encodes a toxin of debatable predictive value, and has been associated with CDI at lower fecal concentrations than other C. difficile strains (24–26). Overall, the patient’s clinical course was consistent with chemotherapy-associated diarrhea, but the timing of CDI treatment onset and improvement in diarrhea confounds the ability to state this conclusively. With these data, we posit that scenario 2 or 3 is more likely than scenario 1. Of note, if this patient did have an initial false-negative EIA for CDI, she did not suffer from any adverse events from delays in initiation of CDI treatment. This EIA^−^/NAAT^+^ patient population remains complex and should be investigated further to understand the predictive value of pathogen (such as the *cdtA-cdtB* locus) and microbiome (C. difficile abundance) markers.

Vancomycin treatment has been reported to select for ARO colonization and shedding in C. difficile patients (18). We examined VRE E. faecium dynamics in patients and found that all of the environmental isolates for vancomycin patients were found at time points after the start of the study drug. Further, SNP analysis indicated that this environmental contamination was associated with patient shedding. Previous observations indicated that vancomycin selects for the presence of VRE populations in patients (18), yet the stool abundance of VREs in those data decreased 2 weeks after treatment; our analyses reveal a different time course of treatment and shedding. In 3 vancomycin-treated patients, E. faecium levels were decreased in the gut during treatment, followed by subsequent E. faecium blooms and patient shedding of VRE strains as far out as 8 weeks after initiation of treatment in some cases. While previous studies have indicated that oral vancomycin could be used prophylactically to prevent CDI (27), our data indicate that it could select for long-term VRE shedding and colonization.

Within this cohort, we examined the effects of vancomycin on patients with EIA^−^/NAAT^+^ stool to better understand the potential risks and benefits of CDI treatment in these patients. The greatest limitation of this study was the small sample size (see Text S1 in the supplemental material). However, one patient randomized to placebo had a subsequently EIA^+^ stool and was started on CDI treatment. An in-depth evaluation of this patient found that an increasing C. difficile burden from enrollment to midtreatment resulted in an EIA^−^ to EIA^+^ status. Conversely, our study failed to demonstrate evidence for oral vancomycin decreasing C. difficile environmental contamination and the potential of transmission, another purported benefit to treating this patient population. Further, vancomycin did shift gut microbial communities, altered the gut resistome, and was associated with environmental contamination by VRE. Based on our observations, the use of oral vancomycin as a prophylaxis may not be beneficial given its short-term effect on C. difficile colonization and its protracted effect on patient shedding of VRE. Additionally, diagnosis of CDI remains a significant clinical challenge; additional work is needed to better define the impact CDI treatment has on EIA^−^/NAAT^+^ patients as well as the development of diagnostics to improve the predictive values of current diagnostics.

## MATERIALS AND METHODS

### Study design.

This study was a double-blinded, randomized controlled trial of 10 days of oral vancomycin (125 mg 4 times per day) versus matching placebo for patients with EIA^−^/NAAT^+^ stool. It was conducted at Barnes Jewish Hospital (BJH) from November 2017 to January 2019.

### Study population.

To be eligible, patients had to be admitted to BJH and have at least one diarrheal stool collected that tested negative for C. difficile toxins via EIA (C. difficile Tox A/B II; Alere, Waltham, MA) by the BJH clinical microbiology laboratory. The stool of potentially eligible patients who did not meet any exclusion criteria (see Text S1 in the supplemental material) was tested by NAAT (Xpert C. difficile; Cepheid, Sunnyvale, CA). Patients whose stool was EIA^−^/NAAT^+^ were approached to participate in the study. All study participants provided written, informed consent. This study (registry number NCT03388268) was approved by the Washington University in St. Louis Institutional Review Board, and the study trial is deposited at https://clinicaltrials.gov/.

### Randomization.

Patients were randomly assigned in a 1:1 ratio to treatment groups using permutation blocks (*n* = 4 per block), stratified by concomitant nonstudy drug antimicrobial use. All study personnel except the study pharmacist were blinded to treatment assignment.

### Study procedure.

The qualifying stool specimen (S00) was originally collected during inpatient clinical care, but patients could complete the study as either inpatient or outpatient participants. After enrollment, patients were contacted daily while on the study drug to determine bowel movement consistency and frequency and any new medication exposures and were assessed for adverse events, including diagnosis of CDI. Stool collection and environmental sampling were performed at enrollment (S01), day 5 (S02), day 10 (S03), week 4 (S04), and week 8 (S05) (Text S1).

### Bacterial culturing and isolate DNA extraction.

To determine a subset of the bacterial microbiota present in each stool specimen, 100 μl of 10-fold dilutions of fecal specimens was plated on blood (BD, Franklin Lakes, NJ) and anaerobic brucella blood (Anaerobe Systems, Morgan Hill, CA) agars for growth of nonfastidious Gram-positive and Gram-negative organisms. Additionally, fecal specimens were plated to MacConkey (MAC) agar with cefotaxime, MAC agar with ciprofloxacin (Hardy Diagnostics, Santa Maria, CA), and VRE ChromID agar (bioMérieux, Durham, NC) to isolate extended-spectrum beta lactamase-producing Gram-negative organisms, Gram-negative quinolone-resistant organisms, and VRE (bioMérieux, Durham, NC), respectively. Plates were incubated at 35°C under aerobic conditions (except in the case of anaerobic brucella blood agar) for 14 h. Individual bacterial colonies from the selective media were identified by MALDI-TOF MS (bioMérieux Vitek MS), and antimicrobial susceptibility testing (AST) was performed using Kirby-Bauer disk diffusion and interpreted using CLSI standards. DNA extraction was performed as previously described (28). C. difficile was cultured anaerobically and isolated (with ribotyping and toxin gene detection performed) as previously described (29, 30). Environmental eswabs were collected using the Eswab specimen collection and transport device; the eswabs were vortexed in eluate briefly, and 100 μl eluate was inoculated on the media described above. Aliquots of isolated organisms were frozen at −80°C in 20% glycerol.

### Library preparation and sequencing of metagenomes and isolate genomes.

Illumina libraries of fecal metagenomic DNA, C. difficile isolate DNA, and VRE isolate DNA were prepared as described previously (28, 31). Reads for both metagenomes and isolate genomes were processed and filtered as described previously (Text S1) (28, 32).

### Metagenomic sequencing analysis.

Species relative abundances were calculated using MetaPhlAn v.2.0 (33). For patients 7, 8, 10, 11, 13, and 15, at time points S00, S03, and S05, resistance gene abundances were calculated using ShortBRED (34) and the comprehensive antibiotic resistance database (CARD) (35). Specifically, *shortbred_quantify.py* was used to determine relative abundances of ARGs based on metagenomics reads. Both species and gene abundances were imported into R using custom Python scripts. The relative abundance of ARG class was calculated as follows. For each patient and each ARG class, ARG read per kilobase per million (RPKM) counts were summed within a class and divided by a patient’s total RPKMs (derived from ShortBRED) to obtain the relative abundance of an antimicrobial resistance gene class. For each group, relative abundances of ARG class were averaged across patients for visualization.

### Whole-genome sequencing and analysis.

Draft genomes were *de novo* assembled into contigs using SPAdes v3.13.0 (36) and assessed for quality using QUAST (37). For downstream analysis, all genome assemblies were required to have <250 contigs and >40× coverage. Using the draft assemblies, we used Prokka v1.12 (38) to annotate C. difficile and VRE genomes. *In silico* multilocus sequence typing (MLST) was performed on all genomes (https://github.com/tseemann/mlst). For each organism, using the .gff files generated by Prokka, a core genome alignment was generated via Roary v3.12.0 (39), and an approximate maximum-likelihood tree was calculated using FastTree v2.1.10 (40). The resulting .newick file was visualized and annotated using iToL (41). VRE isolate genomes were also analyzed by pyANI (https://github.com/widdowquinn/Pyani) to confirm species differences. Resistance genes in VRE isolates were identified by analyzing the Prokka-generated .gff files using AMRfinder (42).

### SNP analysis.

Using the Roary-generated core genome alignments, we computed pairwise SNP distances for all C. difficile and VRE E. faecium isolates using SNP-sites (43). To examine within-patient relatedness, we defined patient groups of strains, whereby all strains (from this study) were the same ST or had <300 SNPs according to our core genome SNP analysis. For each strain group consisting of *n* isolates, we subsampled reads from a given isolate to achieve >75× coverage across the genome. Using these subsampled isolate reads, we generated a pseudoreference assembly for each patient and called SNPs by mapping nonsubsampled quality filtered forward and reverse reads to each assembly using Snippy v4.3.8 (https://github.com/tseemann/snippy). Using the output .bam files, we used Samtools (44) *mpileup -q30* and bcftools view, filtering with *–i 'DP > 10 & QS > 0.95'*, *'FQ<-85' –exclude-types indels*, to generate VCF files for each patient-time point. VCF files were merged, and pairwise SNP distances were computed and visualized in R.

### Data and statistical analysis.

Treatment difference in time to resolution of CSD was computed using a Mann-Whitney U test. Percent change in ARG class abundance was obtained by normalizing relative abundances by the corresponding relative abundance at S00. Statistically significant differences in percent change between vancomycin and placebo groups were determined using a Student's *t* test and corrected for multiple testing using the Holm-Sidak method. For visualization of metagenomics data and computation of community diversity statistics, the packages *phyloseq* and *vegan* were used in R. For visualization of heatmaps concerning AST/ARG profiles, the package *pheatmap* was used. All other statistical tests are described in the figure legends.

### Data availability.

All genomic sequences and metagenomics sequences were deposited in NCBI under BioProject no. PRJNA646752.
