# Supplementary figures and images for: Randomized Controlled Trial of Oral Vancomycin Treatment in Clostridioides difficile-Colonized Patients
Source: mSphere. 2021 Jan 13;6(1):e00936-20. doi: 10.1128/mSphere.00936-20 (PMC7845614; doi:10.1128/mSphere.00936-20)

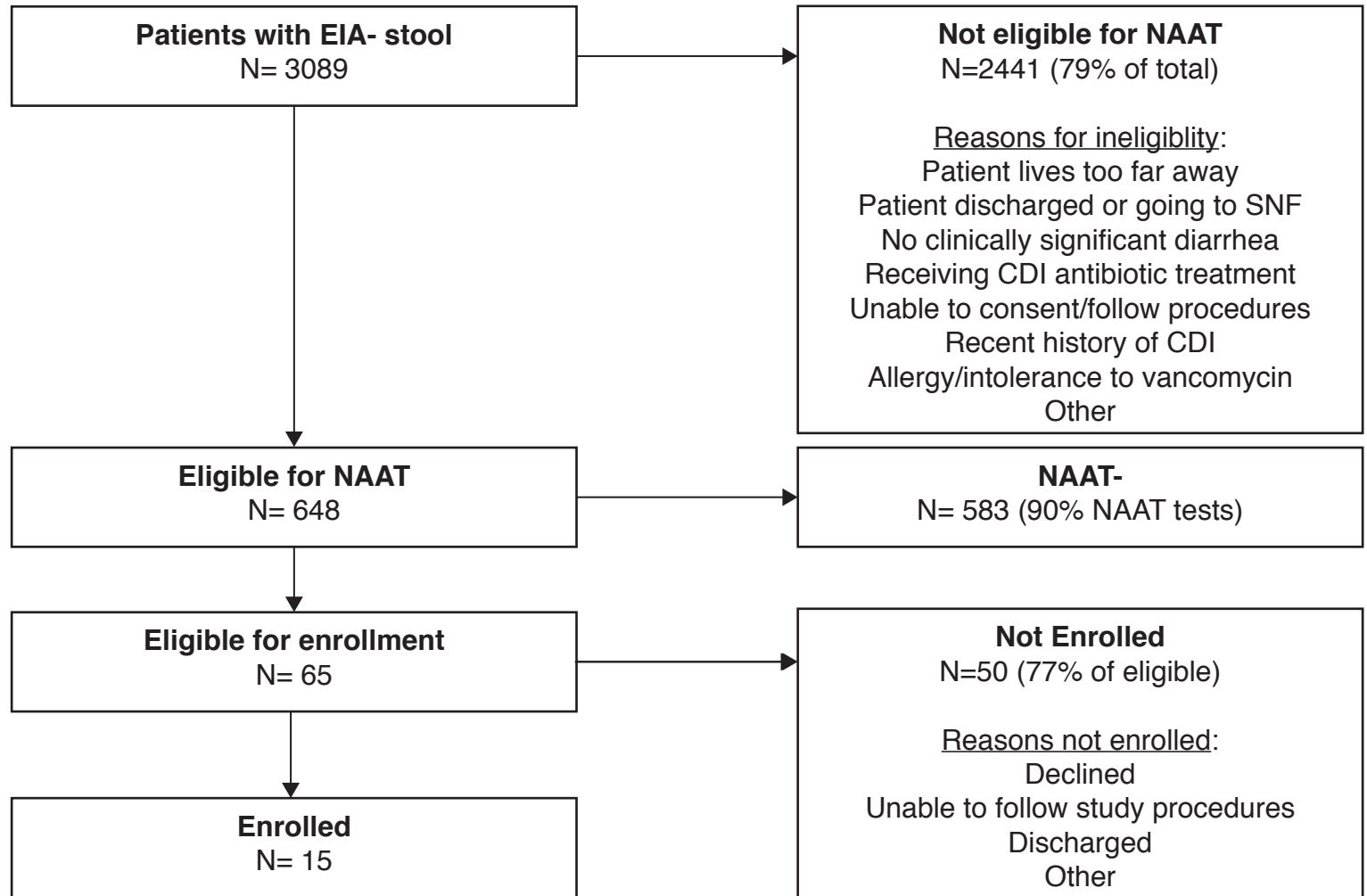

Supplement: FIG S1 [file mSphere.00936-20_sf001.pdf]

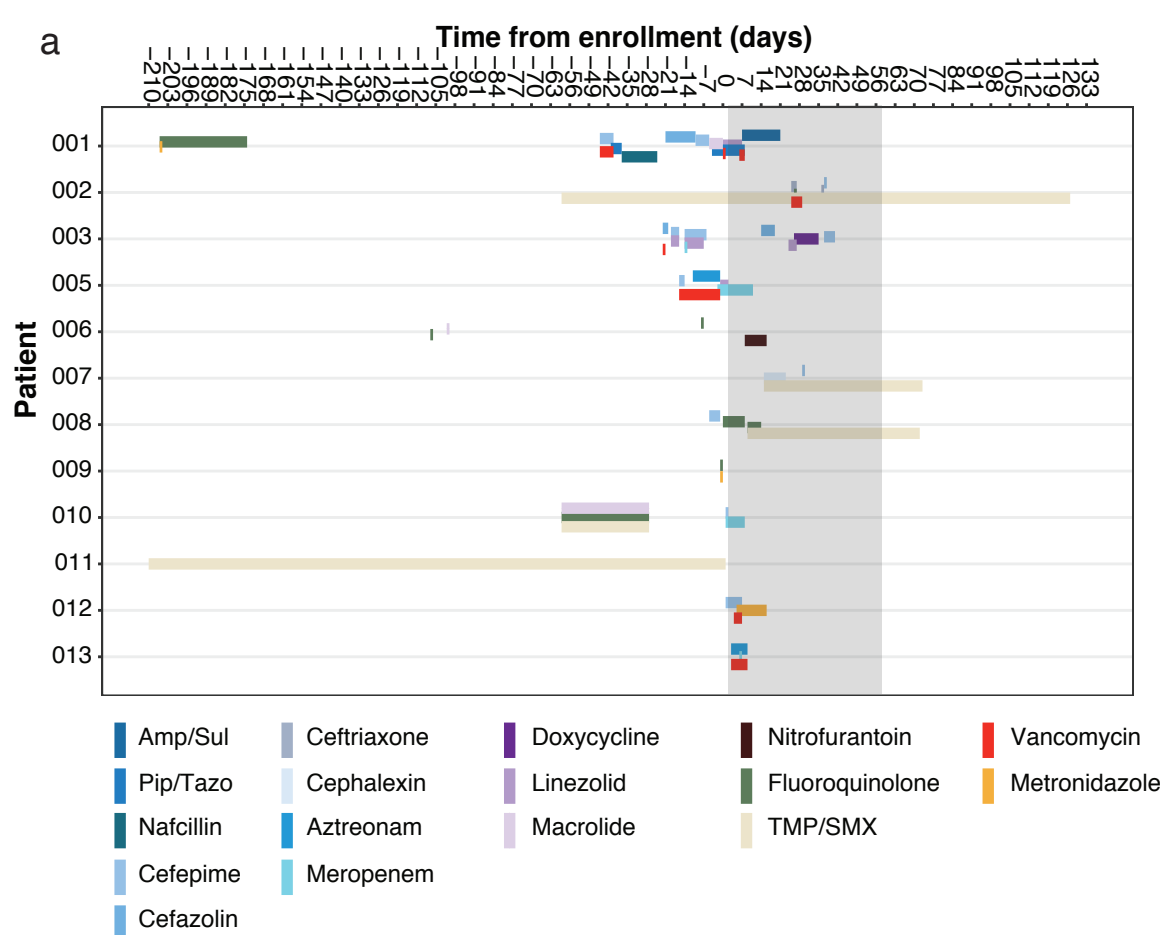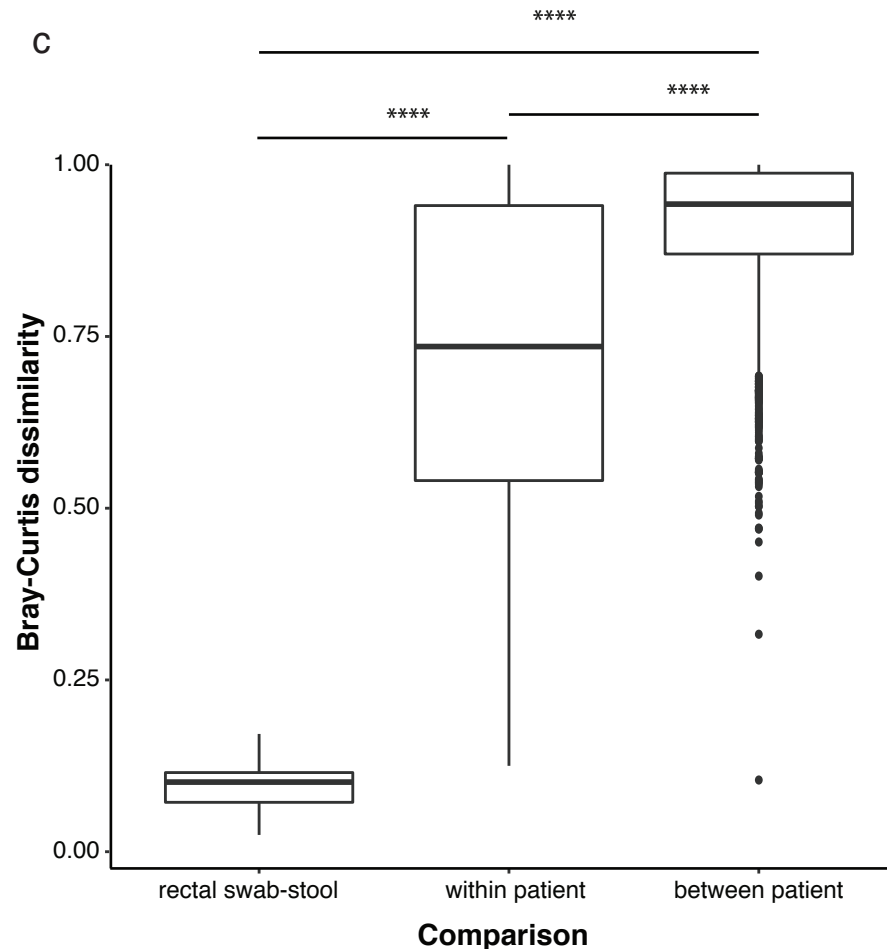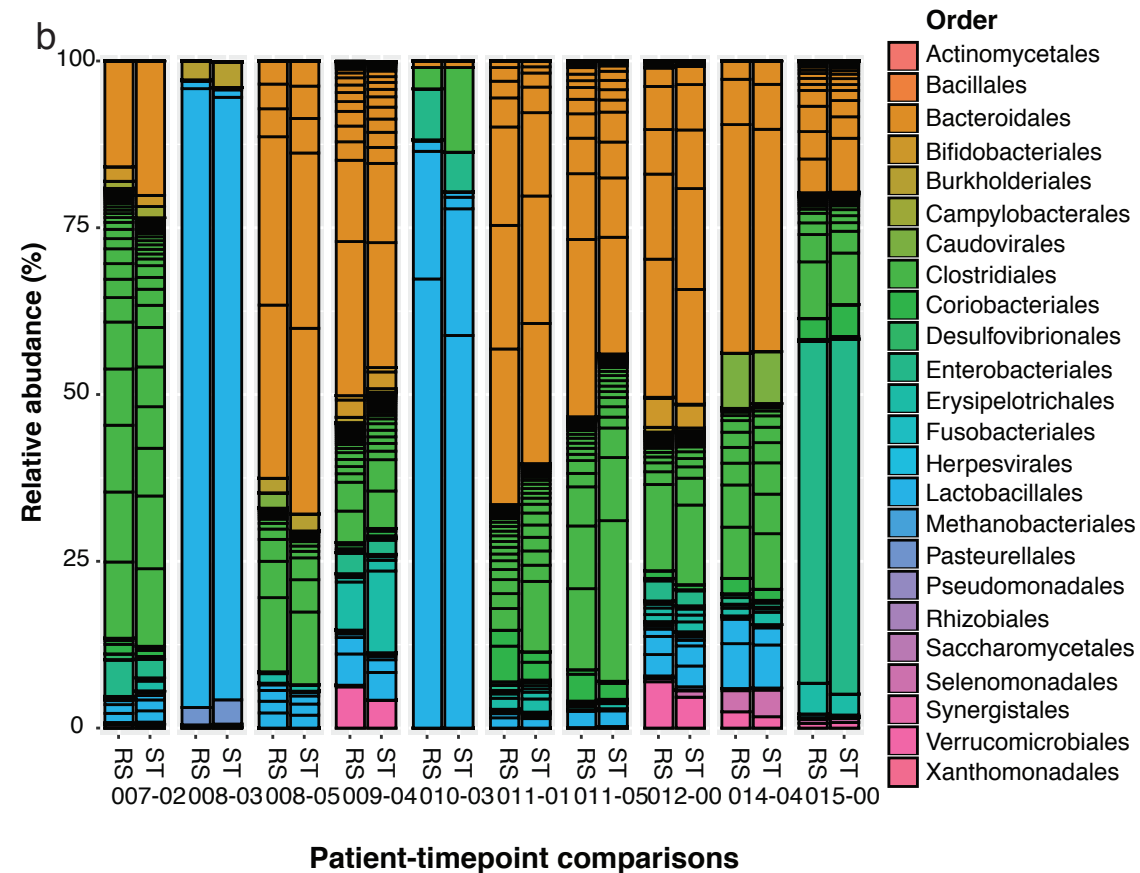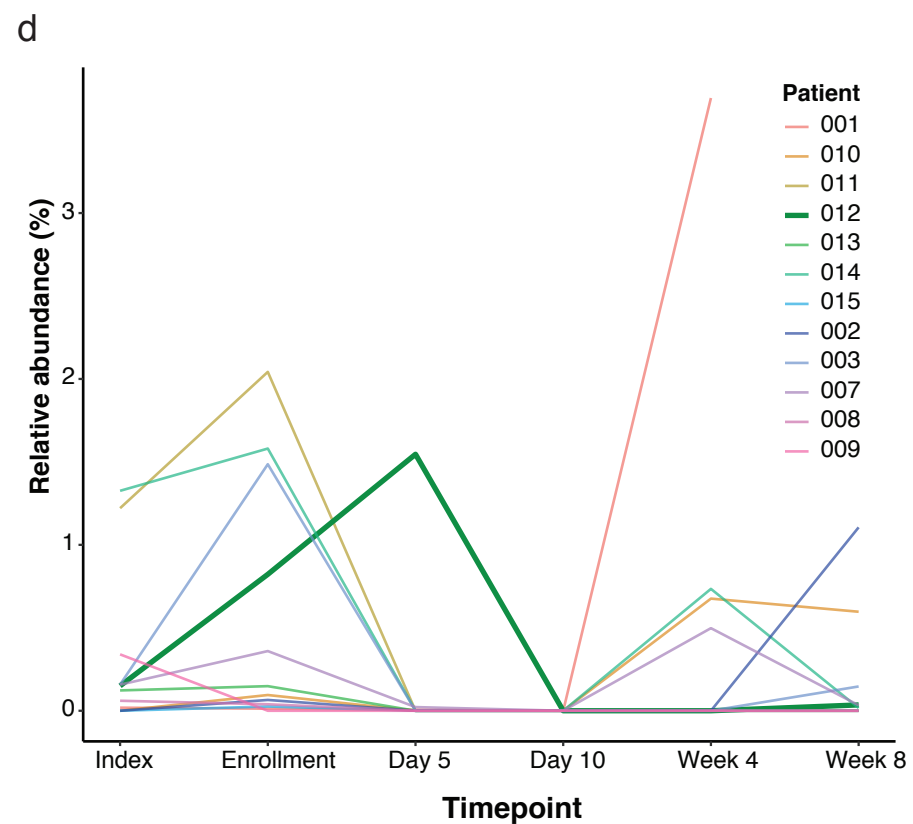

Supplement: FIG S2 [file mSphere.00936-20_sf002.pdf]

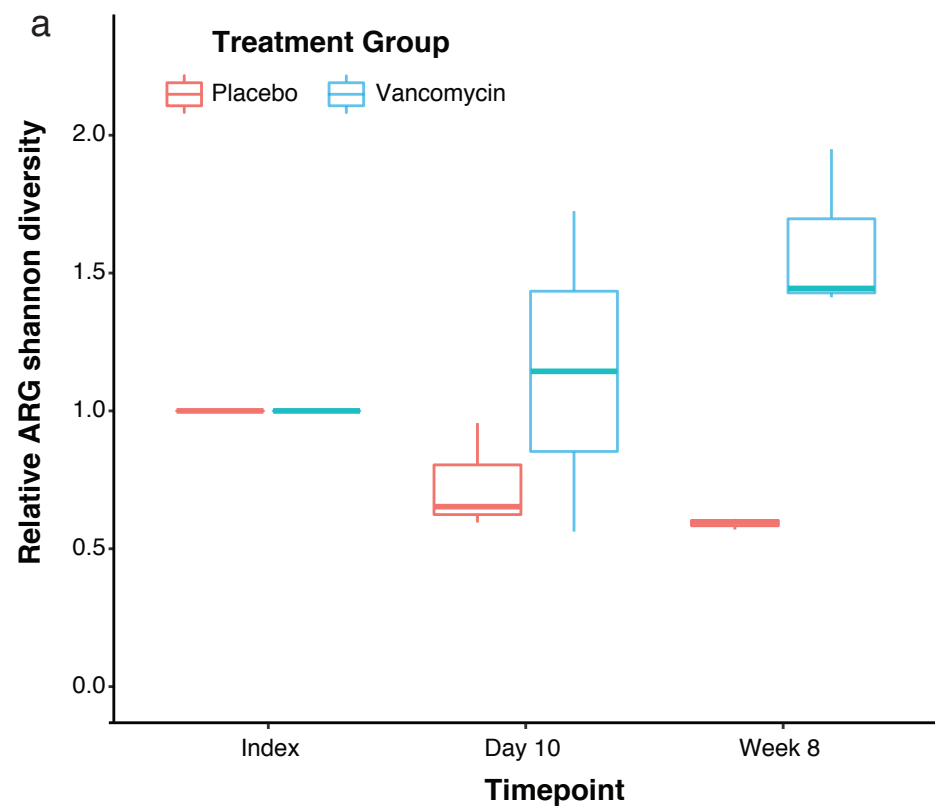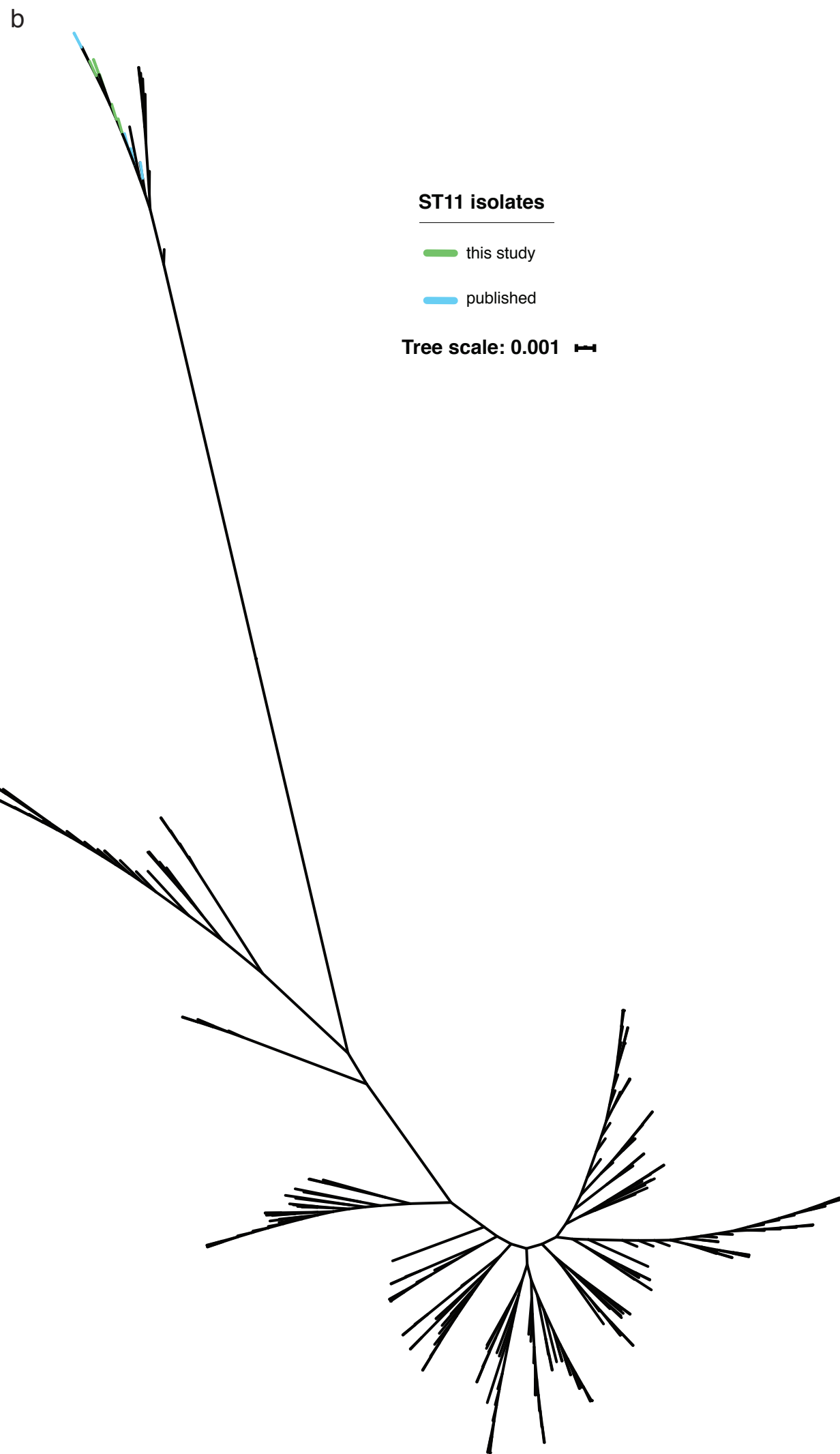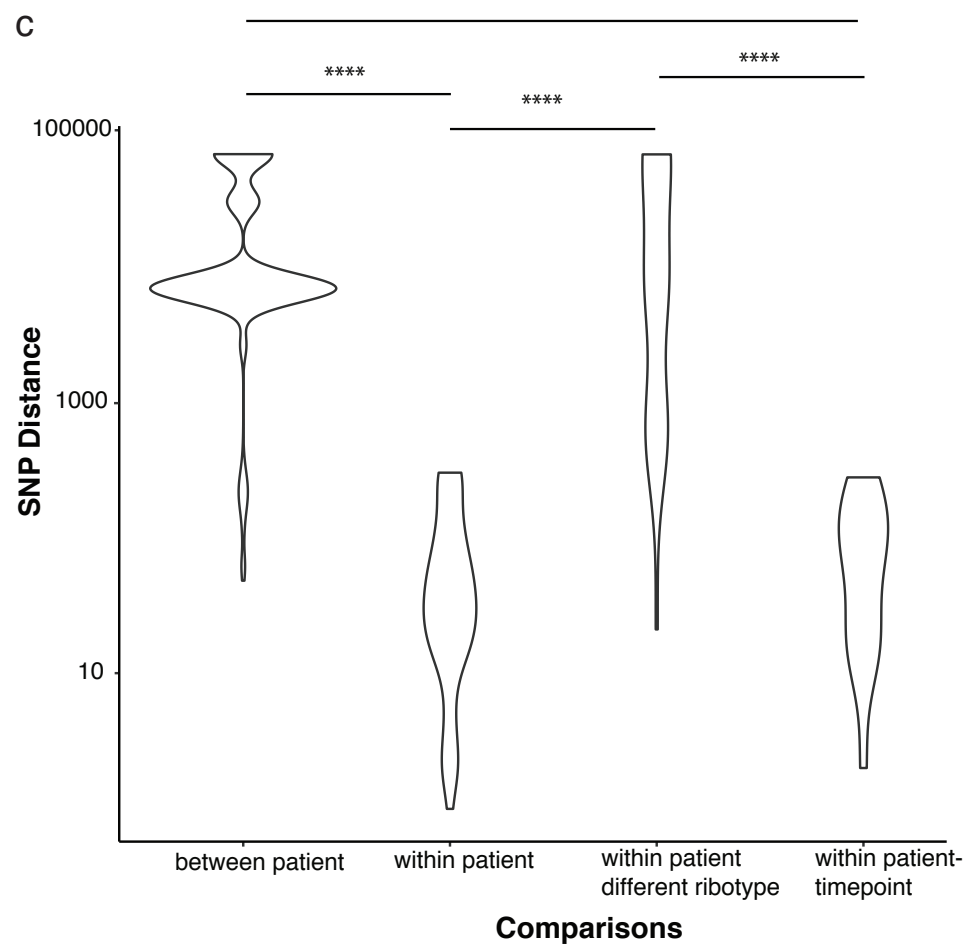

Supplement: FIG S3 [file mSphere.00936-20_sf003.pdf]

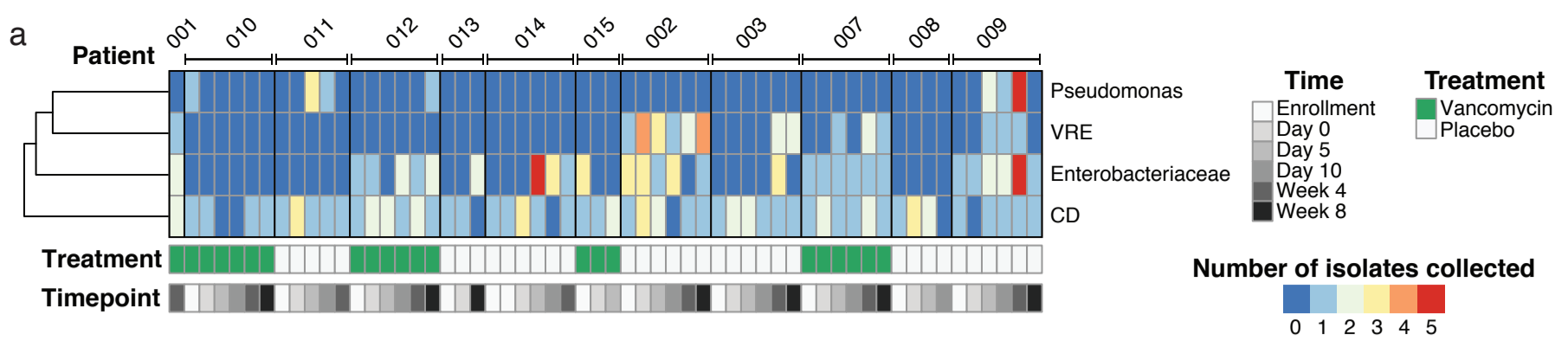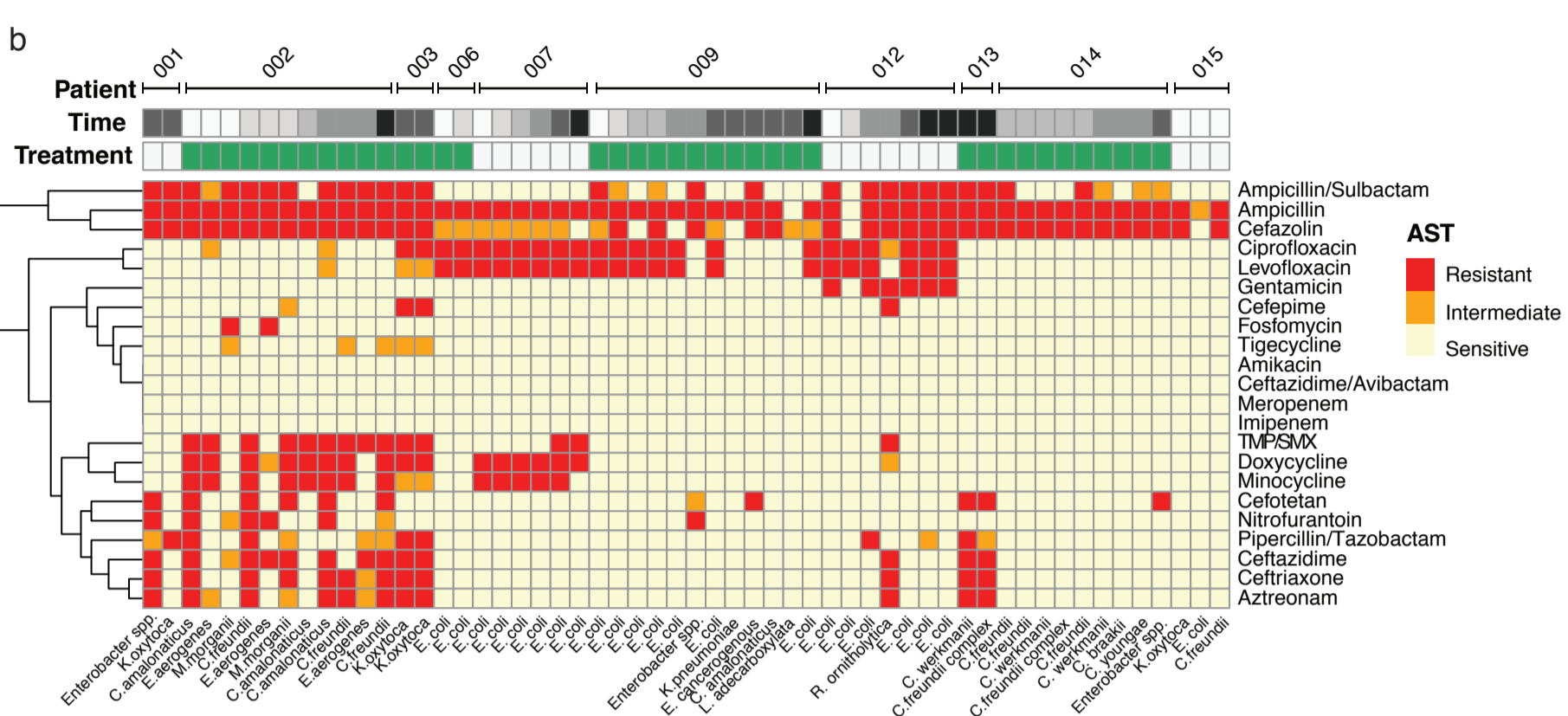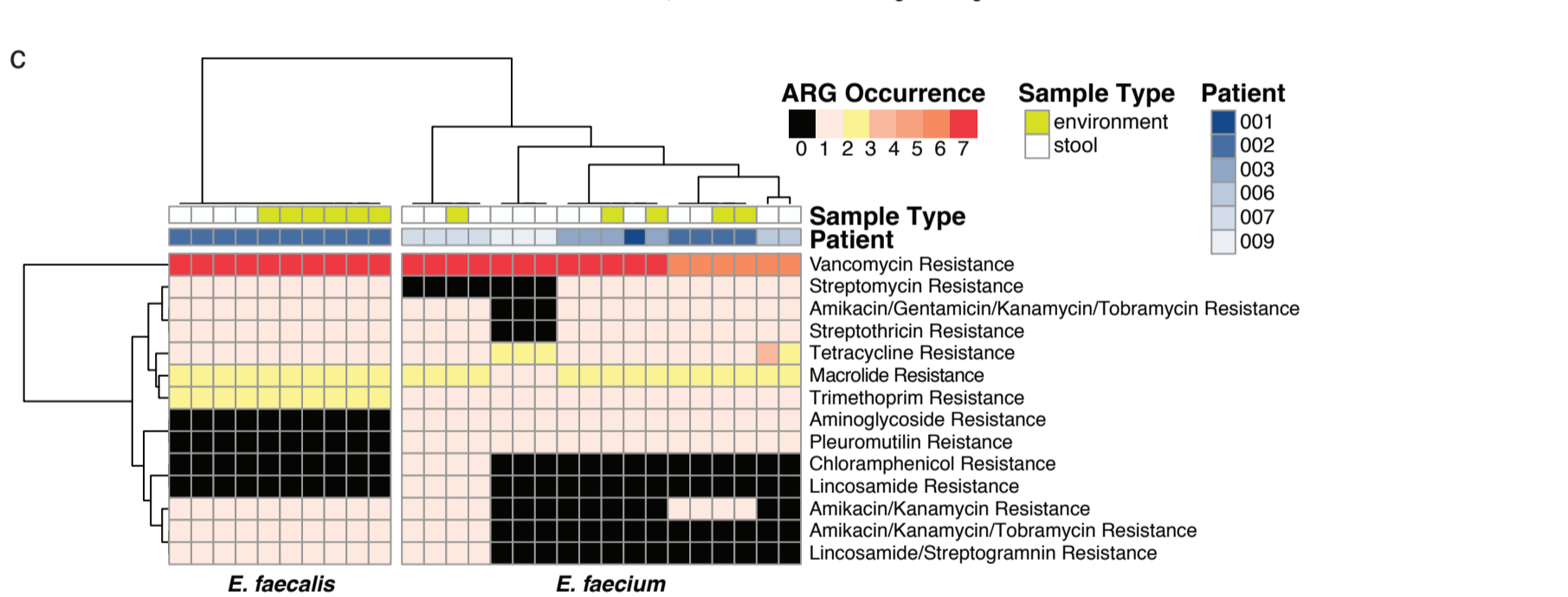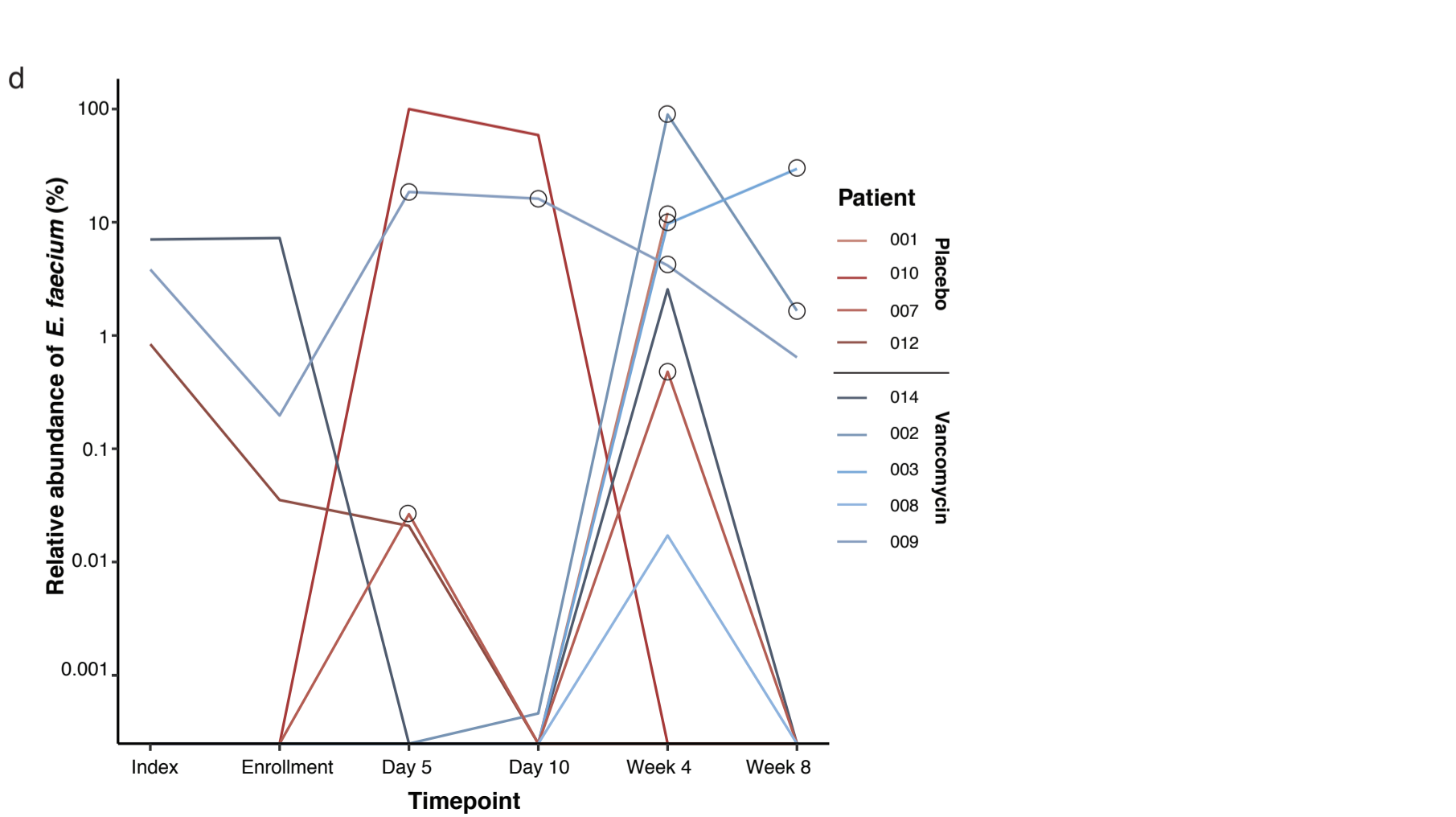

Supplement: FIG S4 [file mSphere.00936-20_sf004.pdf]
