# Supplementary material for: Randomized Controlled Trial of Oral Vancomycin Treatment in Clostridioides difficile-Colonized Patients
Source: mSphere. 2021 Jan 13;6(1):e00936-20. doi: 10.1128/mSphere.00936-20 (PMC7845614; doi:10.1128/mSphere.00936-20)
Supplement: TEXT S1 [file mSphere.00936-20_stxt01.docx]

**Supplementary Material**

**A randomized-controlled trial of oral vancomycin treatment in *C. difficile*-colonized patients**

Skye R. S. Fishbein^1,2,*^, Tiffany Hink^3^, Kimberly A. Reske^3^, Candice Cass^3^, Emily Struttmann^3^, Zainab Hassan Iqbal^3^, Sondra Seiler^3^, Jennie H. Kwon^3^, Carey-Ann D. Burnham^2,4,5^, Gautam Dantas^1,2,4,6,**^, Erik R. Dubberke^3,**^

^1^The Edison Family Center for Genome Sciences and Systems Biology, Washington University School of Medicine, St. Louis, Missouri, USA.

^2^Department of Pathology and Immunology Washington University School of Medicine, St. Louis, Missouri, USA.

^3^Division of Infectious Diseases; Washington University School of Medicine, St. Louis, Missouri, USA

^4^Department of Molecular Microbiology; Washington University School of Medicine, St. Louis, Missouri, USA

^5^Department of Pediatrics, Washington University School of Medicine, St. Louis, Missouri, USA

^6^Department of Biomedical Engineering, Washington University School of Medicine, St. Louis, Missouri, USA

***Correspondence during review**: S.R.S. Fishbein (skye.f@wustl.edu)

**Corresponding authors after review: G. Dantas ([dantas@wustl.edu](mailto:dantas@wustl.edu)); E.R. Dubberke (edubberk@wustl.edu)

**Included in this Document**

**Supplementary Note**

A. Details of the study population

B. Details of the study procedure and bacterial culturing

C. DNA extraction

D. Library preparation and metagenomic sequencing analysis

E. *C. difficile* phylogeny with reference genomes

F. Comparison of stool and rectal swab microbiomes

G. Challenges to study enrollment

**Supplementary References**

Supplementary Note

**A. Details of study population**

Exclusion criteria included: an inability to provide consent, allergy/ intolerance to oral vancomycin, a history of CDI in the past 3 months, not expected to survive though the end of the study follow-up period, a history of receipt of CDI antibiotic treatment, age <=18 years, or that the patient lived too far away to submit specimens to the study team. Enrollment interviews were performed whereby demographics data, bowel movement consistency/frequency, healthcare exposures, dietary preferences, comorbidities, medication exposures, labs, and infection history were collected.

**B. Details of study procedure and bacterial culturing**

All patients were admitted at the time of initial specimen collection. If the patient was hospitalized, stool and environmental samples were collected by the study team. If the patient was unable to have a bowel movement within 24 hours of the timepoint while hospitalized, peri-rectal swabs (RS) were collected using Eswabs (ESwab, Becton, Dickinson, & Company, Franklin Lakes, NJ). For each environmental sample, three pre-moistened flocked swabs (BD, Franklin Lakes, New Jersey) were held together and the surfaces were vigorously sampled. Separate sets of swabs were used for each surface. The following environments were swabbed for patients who were hospitalized: the entire seat of the commode, a 10cm x 10cm area of the bedside table, and a 25cm x 4cm area of the bedrail.

Duration of diarrhea was monitored in patients. Clinically significant diarrhea (CSD) was considered three or more stools in a calendar day. Resolution of CSD was considered two calendar days without diarrhea.

If patients were discharged from the hospital, they were contacted by phone and they had +/- 72 hours to provide a stool sample. For environmental samples of outpatients, the entire seat of the commode, a 10cm x 10cm area of the kitchen counter, and a 10cm x 10cm area of the surface top where most meals are eaten was swabbed. Specimens collected from outpatients were returned to study personnel on the same day as collection. In the case of patients who were too ill to collect specimens, study personnel directly collected specimens from the patient’s residence. To maintain consistency in culture technique, all stool specimens were placed into eswab eluates. The eswab was briefly placed into the stool, immediately placed into the eswab container, and vortexed thoroughly.

**C. DNA extraction**

To extract metagenomics DNA from fecal matter, ~150mg of stool and 200 ul of rectal swab eluate was used as input for the PowerSoil Pro DNA Isolation Kit (Qiagen, Germantown, MD), following manufacturer’s instructions. Sample lysis was modified, and samples were lysed using a Mini-Beadbeater 24 (Biospec Products) with 2 cycles of 2 min at 2500 rpm: 2 min on ice performed. For all isolates of interest, plates corresponding to the bacterial isolate were scraped and genomic DNA was extracted using the QIAamp BiOstic Bacteremia DNA Kit (Qiagen, Germantown, MD) as previously described (1).

**D. Library preparation and metagenomic sequencing analysis**

DNA samples were diluted to 0.5 ng/ul for Illumina library preparation. Illumina libraries were prepared, following the modified Nextera kit protocol (2). Final library concentrations were quantified by Quant-iT PicoGreen (ThermoFisher, Waltham, MA), and three independent pools of all sequencing libraries were created. These pools were combined at equimolar concentration and submitted for 2x150bp paired-end sequencing on an Illumina NextSeq High-Output platform. Libraries were pool and sequenced to achieve (after filtering) 1 million reads for all stool samples and RS samples, 2.5-3.5 million reads for *C. difficile* and VRE isolate genomes, and 5 million reads for a subset of stool samples used for resistome analysis. If a sample had less than 15% bacterial DNA, it was considered unusable for sequencing.

Illumina paired-end reads were binned by index sequences, and reads were trimmed and quality filtered using Trimmomatic v0.38 (3) with the following parameters: *trimmomatic-0.38.jar PE -phred33 ILLUMINACLIP: NexteraPE-PE.fa:2:30:10:1:TRUE LEADING:10 TRAILING:10 SLIDINGWINDOW:4:15 MINLEN:60*. Contaminating human reads were removed using DeconSeq-0.4.3 (*GRCh38* for human)(4). Reads were re-paired using BBtools (5).

**E. *C. difficile* phylogeny with reference genomes**

For the *C. difficile* alignment with publicly available genomes, we downloaded all *C. difficile* genomes from NCBI, and used genomes with <200 contigs. From these genomes, we used MLST as above to determine the representation of strain types (STs) across all genomes. To further filter out non-*C. difficile* genomes, we used only genomes that had >96% nucleotide identity to all other genomes. We selected at least one genome for every strain type and up to 5 genomes for the most abundant strain types. Using these genomes and the isolate genomes from our study, we built a core genome alignment, computed a maximum likelihood tree and visualized it as described in the main text Methods.

**F. Comparison of stool and rectal swab (RS) microbiomes**

Since not all patients provided stool specimens at every timepoint, microbiomes from RS samples and stool samples of the same patient-timepoint were compared to understand congruency between sample types. Qualitatively, relative abundance estimates of taxonomic order composition indicated that RS communities were equivalent to stool communities (Supplementary Figure 2b). Comparison of the beta-diversity (Bray Curtis dissimilarity) between RS and stool samples of the same patient-timepoint relative to other ‘within patient’ distances indicated that these sample types produced highly-related microbiomes (Supplementary Figure 2c; *P*<0.0001). Consequently, one microbiome per patient-timepoint was used (stool if available, otherwise RS).

**G. Challenges to study enrollment**

The original goal was to enroll 50 patients into the trial and this enrollment would have been adequate to assess how vancomycin impacts the microbiome compared to placebo (6). The primary challenge to enrollment was that there were fewer potentially eligible patient than anticipated. We anticipated there would be 1,000 to 1,500 people who would be eligible for this study, but we only identified 648 eligible patients (21% of the 3089 EIA- patients screened). Adding to this challenge, the proportion of EIA- stools that were NAAT+ (6.3%) was also lower than the anticipated 12% (7).

Supplementary References

1. D'Souza AW, Potter RF, Wallace M, Shupe A, Patel S, Sun X, Gul D, Kwon JH, Andleeb S, Burnham CD, Dantas G. 2019. Spatiotemporal dynamics of multidrug resistant bacteria on intensive care unit surfaces. Nat Commun 10:4569.

2. Baym M, Kryazhimskiy S, Lieberman TD, Chung H, Desai MM, Kishony R. 2015. Inexpensive multiplexed library preparation for megabase-sized genomes. PLoS One 10:e0128036.

3. Huch FL. 1973. [Central venous thrombosis and oral contraceptives]. Dtsch Med Wochenschr 98:744.

4. Schmieder R, Edwards R. 2011. Quality control and preprocessing of metagenomic datasets. Bioinformatics 27:863-4.

5. Bushnell B, Rood J, Singer E. 2017. BBMerge - Accurate paired shotgun read merging via overlap. PLoS One 12:e0185056.

6. Isaac S, Scher JU, Djukovic A, Jimenez N, Littman DR, Abramson SB, Pamer EG, Ubeda C. 2017. Short- and long-term effects of oral vancomycin on the human intestinal microbiota. J Antimicrob Chemother 72:128-136.

7. Robinson JI, Weir WH, Crowley JR, Hink T, Reske KA, Kwon JH, Burnham CD, Dubberke ER, Mucha PJ, Henderson JP. 2019. Metabolomic networks connect host-microbiome processes to human Clostridioides difficile infections. J Clin Invest 129:3792-3806.
